# P248: Can hand hygiene and cleaning reduce the number of sick days in day care centers?

**DOI:** 10.1186/2047-2994-2-S1-P248

**Published:** 2013-06-20

**Authors:** T Ibfelt, E Engelund, L Andersen

**Affiliations:** 1 Department of Infection Control, Copenhagen University Hospital, Copenhagen Ø, Denmark; 2 DHI, Hørsholm, Denmark

## Introduction

It is well known that hand hygiene can help reduce the burden of infectious disease both in the community and clinical settings. It is less clear, however, if systematic cleaning and disinfection has the same effect. Young children in daycare centers have a higher amount of days lost to infectious diseases than older children and adults. It is therefore important to know which tools can help reduce the infectious disease pressure in the daycare centers.

## Objectives

To investigate whether cleaning of hotspots or systematic hand hygiene by the staff can reduce the number of days lost to illness among the children in Danish daycare centers (DCC).

## Methods

The study included 23 DCCs with a total of 1703 children. The centers had multiple sections with both nurseries for the youngest children (0-3 years) and kindergartens for children aged 3-6 years. The DCs were divided into 3 intervention groups and a control group: A: Systematic use of hand disinfection by the staff (n=5). B: Systematic cleaning and disinfection of the playroom tables and nursery pillows (n=4). A+B: Both interventions (n=6). 0: Control group (n=8). The interventions were implemented for 3 months during winter 2012. Days of illness were recorded for all children on an individual basis and on a group basis (nursery or kindergarten children).

## Results

The average number of sick for the 3-month period was 2.5 days for all children. The highest number of sick days was seen within the nursery children (mean 3.1 days) while the kindergarten children had fewer sick days (mean 2.1 days).

Hand disinfection by the staff (Intervention A) significantly (p<0.05) reduced the average number of sick days within the kindergarten children by 0,3 days (from 2.1 to 1.8) compared to the control group but did not reduce the number of sick days within the nursery children. Cleaning and disinfection of the nursery pillows (Intervention B) and the combined interventions (A+B) did not reduce the number of sick days within nurseries or kindergarten children.

## Conclusion

There is a potential to reduce the number of sick days by teaching and implementing systematic hand hygiene within the daycare staff, at least in the kindergartens. It also appears that increased cleaning does not affect the number of sick days in neither kindergartens nor nurseries.

## Disclosure of interest

None declared

